# Specialist palliative care teams and characteristics related to referral rate: a national cross-sectional survey among hospitals in the Netherlands

**DOI:** 10.1186/s12904-021-00875-3

**Published:** 2021-11-11

**Authors:** M.S. Boddaert, A. Stoppelenburg, J. Hasselaar, Y.M. van der Linden, K.C.P. Vissers, N.J.H. Raijmakers, L. Brom

**Affiliations:** 1 Netherlands Comprehensive Cancer Organisation (IKNL), P.O. Box 19079, 3501 DB Utrecht, the Netherlands; 2 Netherlands Association for Palliative Care (PZNL), Utrecht, the Netherlands; 3 Center of Expertise in Palliative Care, Leiden University Medical Center, Leiden, the Netherlands; 4 Department of Public Health, Erasmus MC, University Medical Center Rotterdam, Rotterdam, the Netherlands; 5 Department of Anesthesiology, Pain and Palliative Medicine, Radboud University Medical Center, Nijmegen, the Netherlands

**Keywords:** Healthcare delivery, Palliative care, Consultation and referral, Hospitals, Cross-sectional survey

## Abstract

**Background:**

Specialist palliative care teams (SPCTs) in hospitals improve quality of life and satisfaction with care for patients with advanced disease. However, referrals to SPCTs are often limited. To identify areas for improvement of SPCTs’ service penetration, we explored the characteristics and level of integration of palliative care programmes and SPCTs in Dutch hospitals and we assessed the relation between these characteristics and specialist palliative care referral rates.

**Methods:**

We performed a secondary analysis of a national cross-sectional survey conducted among hospitals in the Netherlands from March through May 2018. For this survey, a previously developed online questionnaire, containing 6 consensus-based integration indicators, was sent to palliative care programme leaders in all 78 hospitals. For referral rate we calculated the number of annual inpatient referrals to the SPCT as a percentage of the number of total annual hospital admissions. Referral rate was dichotomized into high (≥ third quartile) and low (< third quartile). Characteristics of SPCTs with high and low referral rate were compared using univariate analyses. *P*-values < 0.05 were considered significant.

**Results:**

In total, 63 hospitals (81%) participated in the survey, of which 62 had an operational SPCT. The palliative care programmes of these hospitals consisted of inpatient consultation services (94%), interdisciplinary staffing (61%), outpatient clinics (45%), dedicated acute care beds (21%) and community-based palliative care (27%). The median referral rate was 0.56% (IQR 0.23–1.0%), ranging from 0 to 3.7%. Comparing SPCTs with high referral rate (≥1%, *n* = 17) and low referral rate (< 1%, *n* = 45) showed significant differences for SPCTs’ years of existence, staffing, their level of education, participation in other departments’ team meetings, provision of education and conducting research. With regard to integration, significant differences were found for the presence of outpatient clinics and timing of referrals.

**Conclusion:**

In the Netherlands, palliative care programmes and specialist palliative care teams in hospitals vary in their level of integration and development, with more mature teams showing higher referral rates. Appropriate staffing, dedicated outpatient clinics, education and research appear means to improve service penetration and timing of referral for patients with advanced diseases.

**Supplementary Information:**

The online version contains supplementary material available at 10.1186/s12904-021-00875-3.

## Introduction

Early provision of specialist palliative care for patients with advanced disease or frailty strongly relates to a better quality of life, less depression and anxiety and higher satisfaction with care [1–6]. Moreover, population-based cohort studies across various care settings have also shown better quality of care, defined as less potentially inappropriate healthcare utilization at the end of life, in patients who received early specialist palliative care [7–10]. Consequently, international guidelines recommend early integration of specialist palliative care and oncology care [11, 12].

Based on international expert consensus, 13 major integration indicators have been developed to assess the level of integration of specialist palliative care and oncology care in hospitals [13]. A survey study using these indicators demonstrated that European Society for Medical Oncology (ESMO) - Designated Centres (DCs) of Integrated Oncology and Palliative Care (PC) had high levels of integration with regard to palliative care programme organisation, but timing of referral and education remained areas for further development [14].

Among multiple models of palliative care delivery, hospital-based specialist palliative care teams (SPCTs) are one way to promote the integration of patient-directed care and disease-directed treatment [15–17]. As a measure for SPCTs’ service penetration, the National Palliative Care Registry in the United States used the number of annual inpatient palliative care consultations as a percentage of the number of total annual hospital admissions to determine referral rate [18]. Over the years they have reported a steadily increasing average referral rate [19]. Moreover, they demonstrated that higher staffing levels of SPCTs were associated with higher referral rates, which were subsequently associated with earlier initial palliative care consultation during hospital admission [18].

In the Netherlands all healthcare professionals are expected to provide generalist palliative care, informed by national standards and guidelines. Palliative care specialists can be consulted to provide support and expert advice. As such, professional oncology standards, issued by the Dutch Federation of Oncological Societies (SONCOS), state that from 2017 every hospital providing cancer care should have a SPCT available [20]. Staffing of the SPCT should at least consist of 2 medical specialists and a nurse with specific expertise in palliative care. The nurse is preferably an oncology nurse or nurse practitioner in oncology or anaesthesiology/pain medicine. At least one of the medical specialists must have completed specific training in the field of palliative care.

In a three-yearly survey study we routinely monitor the development of SPCTs and explore their characteristics [21]. Although our survey study in 2015 showed an increase in SPCTs in Dutch hospitals, the number of inpatient palliative care referrals was limited (median 77 consultations per year) and the referral rate was low (mean 0.6%) compared to hospitals in the US (mean 4.4%) [18, 21]. Additionally, we demonstrated a wide variation in interdisciplinary staffing of SPCTs, level of education and experience. We conducted a secondary analysis of the survey study in 2018 to specifically explore characteristics and level of integration of palliative care programmes and their SPCTs as well as the relation between these characteristics and high or low specialist palliative care referral rates, to identify areas for improvement of SPCTs’ service penetration. We hypothesized that hospital palliative care programmes with high referral rates would be better staffed and better integrated, with earlier timing of referrals.

## Methods

### Study design

This is a secondary analysis to identify characteristics of hospital palliative care programmes and their SPCTs related to higher referral rates based on the results of a three-yearly cross-sectional survey of SPCTs in all Dutch hospitals, conducted from March through May 2018. The STROBE reporting guideline for cross-sectional studies was used [22]. Results from the primary analysis have been described elsewhere [23].

### Setting and participants

The heads of SPCTs or palliative care programme leaders of all 78 hospitals in the Netherlands were invited to participate in a voluntary online survey. The hospitals consisted of general (*n* = 38), teaching (*n* = 30) and university (*n* = 8) hospitals and dedicated oncology centres (*n* = 2). For the online questionnaire, Survey Monkey was used and to maximize response rate, the participants were sent a reminder after 2 weeks. No financial incentives were provided.

### Questionnaire

For the first survey in 2013, we generated survey questions based on interviews and expert meetings [24]. Subsequently, the questionnaire for the second survey in 2015 was pilot tested by several SPCT members for face validity and readability, and length of the questionnaire [21]. The questionnaire for the survey in 2018 was reviewed and updated to serve the primary aim of this three-yearly survey focused on the development and characteristics of SPCTs in Dutch hospitals. The final version of the (online) questionnaire contained 77 items based on the Donabedian triad of structure, processes and outcomes (see Additional file 1) [25].

The first part of the questionnaire focused on hospital and palliative care programme characteristics, including assignment of executive board, year of start of SPCT, presence of inpatient consultation services, of dedicated outpatient clinics, of dedicated acute care beds, of community-based palliative care, of triggered referral for specific diagnoses, and timing of referrals. Community-based palliative care was defined as providing bedside consultation at home and having professionals from both hospital and primary care setting on the SPCT.

The second part of the questionnaire assessed SPCT characteristics including staffing, level of education, process of consultation, didactic and research efforts and numbers of consultations in various settings.

With regard to level of education, professional oncology standards recommend that SPCT members are trained in specialist palliative care [20]. As in the Netherlands palliative medicine is not a certified medical or nursing (sub)specialty, the questionnaire listed the available postgraduate palliative care education for both physicians, nurses and nurse practitioners to assess the level of education of SPCT members. This included an 8-day medical course and a 2- year continuing medical education (CME) for physicians, a basic palliative care training and a 1-year continuing nursing education (CNE) for nurses and a differentiation in palliative care for nurse practitioners.

### Indicators to assess integration of palliative care in hospital care

To evaluate hospital-wide integration of palliative care we used six indicators from an existing set of 13 major indicators for integration of oncology and palliative care programmes, established through international expert consensus [13]. Our limitation to six indicators was pragmatically based on availability of items in the questionnaire that was developed for the three-yearly survey study. The six indicators available from our survey were: presence of inpatient consultation services, presence of a dedicated outpatient clinic, interdisciplinary staffing of the SPCT (i.e. including at least a physician, nurse and psychosocial team member such as psychologist/counsellor, chaplain, social worker), routine symptom screening of palliative care patients, early referral to SPCT, and presence of a didactic palliative care curriculum. Three indicators were adjusted to the Dutch hospital setting. First, routine symptom screening of palliative care patients was broadened to make it suitable for the evaluation of hospital wide integration of palliative care. We considered the use of a tool for identification of palliative care patients to be similarly suitable as routine symptom screening. Second, early referral to SPCT was defined as a need-based referral > 3 months before death, based on previous literature, as international consensus on the definition of early referral is still lacking [13, 26].

Last, the indicator ‘evaluating availability of a didactic palliative care curriculum for fellows in oncology’ was broadened to the availability of a palliative care curriculum for nurses, interns, residents and/or fellows throughout the hospital.

### Statistical analysis

Descriptive statistics were used to summarize characteristics of the hospital palliative care programmes, SPCTs and integration indicators. Hospitals without an operational SPCT were excluded from the analysis. Characteristics of the SPCTs were described for teams with inpatient referrals (i.e., referral rate > 0%). As each palliative care programme could score multiple integration indicators, we calculated an integration index. This represents a composite score of the six integration indicators; 1 point was given for each affirmative response. Total score ranges from 0 to 6, with a higher index indicating a greater level of integration.

To assess relations between characteristics and high specialist palliative care referral rates, we determined referral rates by calculating the number of annual inpatient palliative care consultations as a percentage of total annual hospital admissions [18]. We calculated mean, range, median and interquartile range (IQR) of referral rates to detect if results had a skewed distribution. To differentiate between teams with high and low referral rates, we used the IQR and defined referral rate as low for SPCTs with referral rate < third quartile and as high for SPCTs with referral rate ≥ third quartile.

SPCTs with high and low referral rate were compared in univariate analysis using t-test, Chi-square test and Fisher exact test. Missing data > 5% were reported. All analyses were two-sided and *p* values < 0.05 were considered significant. All analyses were conducted using STATA version 16.1 (StataCorp LLC, Texas, USA).

To evaluate representability of our study population, all non-responding hospitals were contacted after the survey closed to verify hospital type, presence of an operational SPCT and certification as ESMO-DC of integrated oncology and PC.

## Results

In all, 63 out of 78 Dutch hospitals participated, resulting in a response rate of 81%. Respondents consisted of 27 general hospitals, 26 teaching hospitals, eight university hospitals and two dedicated oncology centres. All but one general hospital had an operational SPCT and thus 52 non-tertiary (general and teaching hospitals) and 10 tertiary hospitals (university hospitals and dedicated oncology centres) were included for analysis (Table 1).

**Table 1:** Characteristics of hospitals and their palliative care programme stratified by referral rate^a^

	Total (***n*** = 62)	Low referral rate^**a**^ (***n*** = 45)	High referral rate^**a**^ (***n*** = 17)	*P*-value
**Number of hospital admissions / year** *(mean, SD)*	23.622 (11.856)	23.813 (12.626)	23.116 (9.853)	.84
	**N (%)**	**N (%)**	**N (%)**	
**Type of hospital**				.33
*Tertiary*	10 (16)	6 (13)	4 (24)	
*Non-tertiary*	52 (84)	39 (87)	13 (76)	
**ESMO-DC of integrated oncology and PC**	13 (21)	8 (18)	5 (29)	.32
**PC assignment of the hospital executive board**	37 (60)	27 (60)	10 (59)	.93
**Existence of specialist palliative care team** ^b^				**.04**
*≤ 3 years*	24 (39)	21 (47)	3 (18)	
*> 3 years*	38 (61)	24 (53)	14 (82)	
**Presence of inpatient PC consultation services**	58 (94)	41 (91)	17 (100)	.57
**Presence of dedicated PC outpatient clinic**	28 (45)	15 (33)	13 (76)	**.004**
**Presence of dedicated acute care beds**	13 (21)	10 (22)	3 (18)	1.00
**Presence of physical dedicated PC unit (***n* = 13)	6 (46)	5 (50)	1 (33)	.61
**Provision of community-based palliative care**^c^	17 (27)	10 (22)	7 (41)	.14
**Routine identification of PC patients**^d^	36 (58)	22 (51)	13 (76)	.09
**Triggered referral for specific diagnoses**	12 (19)	8 (18)	4 (24)	.72
**Average timing of referral prior to death**				**< .001**
< 3 days	5 (8)	5 (11)	0	
4 days – 2 wks	19 (31)	18 (40)	1 (6)	
2 wks – 4 wks	10 (16)	9 (20)	1 (6)	
4 wks- 3 months	19 (31)	7 (16)	12 (71)	
> 3 months	2 (3)	2 (4)	0	
Unknown	7 (11)	4 (9)	3 (18)	

^a^Referral rate: N^o^ of annual inpatient referrals / N^o^ of total annual hospital admissions × 100. Low referral rate < 1% (third quartile), high referral rate ≥ 1%. ^**b**^The cut off at 3 years existence was based on previous research of Brinkman et al. showing a difference in referral rate between SPCTs younger and older than 3 years [21]. ^c^Community-based palliative care defined as providing bedside consultation at home and having professionals from both hospital and primary care setting on the SPCT. ^d^ use of **t**ool for identification of palliative care patients

Non-responding hospitals consisted of 11 general hospitals and 4 teaching hospitals, of which one was certified as ESMO-DC of integrated oncology and PC. All non-responding hospitals had an operational SPCT.

### Hospital and palliative care programme characteristics

In total, 94% (*n* = 58) of all 62 hospitals provided inpatient palliative care (PC) consultation services, 45% (*n* = 28) offered dedicated outpatient clinics, 21% had dedicated acute care beds present and 27% (*n* = 17) provided community-based palliative care (Table 1). Most palliative care programmes had an assignment of the executive board (60%, *n* = 37) and most SPCTs had been operational for over 3 years (61%, *n* = 38). Thirteen hospitals (21%) were certified as an ESMO-DC of integrated oncology and PC. Additionally, more than half of the hospitals (58%, *n* = 36) routinely used a tool to identify palliative care patients. In 31% (*n* = 19) referrals mostly occurred in the last 4 days to 2 weeks before death and in 31% (n = 19) referrals mostly took place in the last 4 weeks to 3 months before death. Triggered referrals for specific diagnoses occurred in 19% (*n* = 12) of the palliative care programmes.

### Hospital and palliative care programme characteristics stratified by referral rate

Referral rates to specialist palliative care ranged from 0 to 3.7% with a mean of 0.85%, a median of 0.56% and an IQR between 0.23–1.0%. Defined by a cut-off at the third quartile, 45 hospital palliative care programmes had a low (< 1%) and 17 had a high referral rate (≥ 1%).

In palliative care programmes with high referral rates, SPCTs more often existed longer than 3 years compared to SPCTs in programmes with low referral rates (82% vs 53%; *p* = 0.04) (Table 1) and dedicated outpatient clinics were present more often (76% vs 33%; *p* = 0.004). Timing of referral also differed: in high referral rate programmes most patients were referred between 4 weeks to 3 months before death (71%), while in low referral rate programmes referrals mostly occurred in the last 4 days to 2 weeks before death (40%)(*p* < 0.001).

### Specialist palliative care team characteristics

In total, 58 SPCTs had inpatient consultation services and their characteristics are presented below. Four teams had no inpatient palliative care referrals (referral rate was 0%), they had been operational for less than 3 years.

On average these 58 SPCTs were staffed with 4.3 (SD 2.4) physicians and 2.3 (SD 2.4) nurses and they had a mean of respectively 13.2 (SD 16.4) and 35.8 (SD 27.9) designated hours per week to participate in their SPCT (Table 2). Of all 58 SPCTs, 36 had designated hours for a psychologist / counsellor, chaplain or social worker (mean designated hours was 1.2 (SD 3.0)). Two thirds of SPCTs had at least one physician with a 2-year PC continuing medical education (CME) (67%, *n* = 39), nurse with a 1-year PC continuing nursing education (CNE) (66%, *n* = 38)) or nurse practitioner (71%, *n* = 41) on their team. Additionally, 38% (*n* = 22) of SPCTs employed nurses with basic palliative care training.

**Table 2:** Characteristics of specialist palliative care teams stratified by referral rate^a^

	Total (n = 58)	Low referral rate^a^ (n = 41)	High referral rate^a^ (n = 17)	*P*-value
	**Mean (SD)**	**Mean (SD)**	**Mean (SD)**	
**N**^**o**^ **of inpatient referrals**	202 (177)	114 (78)	417 (168)	
**N**^**o**^ **of outpatient referrals** (n = 35^b^)	65 (96)	29 (55)	120 (119)	**.004**
**N**^**o**^ **of community visits** (n = 17)	12 (18)	6 (12)	23 (24)	.06
**SPCT Staffing**				
N^o^ of physicians in team	4.3 (2.4)	4.3 (2.4)	4.5 (2.5)	.71
N^o^ of nurses in team	2.3 (2.4)	2.2 (2.3)	2.7 (2.6)	.44
N^o^ of designated PC hours / w (physicians)	13.2 (16.4)	9.2 (12.3)	22.8 (20.9)	**.003**
N^o^ of designated PC hours / w (nurses)	35.8 (27.9)	29.3 (24.9)	51.5 (29.3)	**.004**
N^o^ of designated PC hours / w (psychosocial)^f^ (n = 36)	1.2 (3.0)	1.0. (2.5)	2.3 (4.5)	.29
**N**^**o**^ **of team members with specific training**				
# physicians with 2-year palliative care CME^d^	1.3 (1.3)	1.1 (1.1)	1.7 (1.7)	.12
# physicians with 8-day course in palliative care	2.5 (2.2)	2.5 (2.0)	2.7 (2.6)	.70
# nurses with 1-year palliative care CNE^e^	1.6 (1.8)	1.2 (1.6)	2.4 (2.1)	**.022**
# nurses with basic PC training	0.7 (1.4)	0.9 (1.5)	0.3 (1.0)	. 12
# nurse practitioner	1.5 (1.4)	1.3 (1.4)	1.9 (1.5)	.17
	**N (%)**	**N (%)**	**N (%)**	
**Level of education present in team**				
Physician(s) with 2-year palliative care CME^d^	39 (67)	26 (63)	13 (76)	.38
Physician(s) with 8-day course in palliative care	58 (100)	41 (100)	17 (100)	1.00
Nurses with 1-year palliative care CNE^e^	38 (66)	25 (61)	13 (76)	.37
Nurses with basic PC training	22 (38)	20 (49)	2 (12)	**.009**
Nurse practitioner PC	41 (71)	27 (66)	14 (82)	.34
**Participation in other departments’ MDTMs**^c^	30 (52)	17 (41)	13 (76)	**.021**
**Availability outside office hours**	10 (17)	7 (20)	2 (12)	.79
**Initial consultation**				.69
Nurse-based	36 (62)	24 (59)	12 (71)	
Physician-based	21 (36)	16 (39)	5 (29)	
Unknown	1 (2)	1 (2)	–	
**Proportion of non-oncology referrals**				.20
< 20%	14 (24)	11 (27)	3 (18)	
20–40%	27 (47)	15 (37)	12 (71)	
40–60%	13 (22)	11 (27)	2 (12)	
60–80%	3 (5)	3 (7)	0	
> 80%	1 (2)	1 (2)	0	
**Non-clinical activities**				
Education inside own hospital	57 (98)	40 (98)	17 (100)	1.00
Education outside own hospital	41 (71)	24 (59)	17 (100)	**.001**
Research	22 (38)	11 (27)	11 (65)	**.016**

^a^Referral rate: N^o^ of inpatient referrals / N^o^ of hospital admissions × 100. Low referral rate < 1% (third quartile), high referral rate ≥ 1%. ^b^Not all SPCTs had a dedicated outpatient clinic, while providing out-patient consultations; ^c^*MDTM* Multidisciplinary team meeting ^d^ *CME* Continuing medical education; ^e^ *CNE* Continuing nursing education. ^f^Psychologist / counsellor, chaplain, social worker

Half of all SPCTs (52%, *n* = 30) participated in other departments’ multidisciplinary team meetings (MDTMs) and 17% (*n* = 10) of SPCTs were available outside office hours.

Most SPCTs provided nurse-based initial consultation (62%, *n* = 36). Overall, SPCTs had a mean annual number of inpatient referrals of 202 (SD 177). For 35 SPCTs providing outpatient care the mean number of outpatient referrals was 65 (SD 96). Of all SPCTs 17 provided consultations in the community, with a mean of 12 (SD 18) visits per year. The largest group of SPCTs (47%, *n* = 27) indicated that the proportion of non-oncology referrals was between 20 and 40%.

Almost all SPCTs (98%, *n* = 57) provided education within their own hospital and 71% (*n* = 41) provided education outside their own hospital. Over one third of all SPCTs participated in research (38%, *n* = 22).

### Specialist palliative care team characteristics stratified by referral rate

Of all 58 SPCTs, 41 had a low referral rate and 17 had a high referral rate.

High referral rate SPCTs had more designated hours per week for both physicians (22.8 vs 9.2; *p* = 0.003) and nurses (51.5 vs 29.3; *p* = 0.004) compared to low referral rate SPCTs (Table 2). Also, high referral rate SPCTs employed more nurses with a 1-year PC CNE compared to low referral rate SPCTs (2.4 vs 1.2; *p* = 0.022), whereas low referral rate SPCTs more often included nurses with basic PC training compared to high referral rate SPCTs, respectively 49% vs. 12% (*p* = 0.009).

High referral rate SPCTs more often participated in multidisciplinary team meetings than low referral rate SPCTs (76% vs 41%; *p* = 0.021).

The mean number of annual inpatient referrals was 114 (SD 78) for low referral rate SPCTs and 417 (SD 168) for high referral rate SPCTs. Similarly, SPCTs with low referral rate provided less outpatient consultations than SPCTs with high referral rate, respectively 29 (SD 55) vs 120 (SD 119) (*p* = 0.004). Education outside their own hospital was provided by all high referral rate SPCTs and by 59% of low referral rate SPCTs (*p* = 0.001). Also participation in research differed between teams with high and low referral rates, respectively 65 and 27% (*p* = 0.016).

### Level of hospital-wide integration of specialist palliative care

Evaluation of hospital-wide integration of specialist palliative care programmes by use of the six integration indicators, showed that 94% (*n* = 58) of all 62 hospitals provided inpatient consultation services, 45% (*n* = 28) had outpatient clinics and 61% (*n* = 38) had interdisciplinary staffing of the SPCTs. Also, more than half of the hospitals (58%, *n* = 36) routinely used a tool to identify palliative care patients, 3% (n = 2) on average referred patients to SPCTs more than 3 months before their death and most hospitals (95%, *n* = 59) had a didactic palliative care curriculum (Table 3). The integration index resulted in a higher, near significant level of integration for high referral rate palliative care programmes compared to low referral rate programmes (3.42 vs 3.94; *p* = 0.06).

**Table 3:** Level of hospital-wide integration of specialist palliative care (adapted from Hui et al. 2015)

	Total (n = 62)	Low referral rate^a^ (n = 45)	High referral rate^a^ (n = 17)	*P*-value
**Integration indicators**	**N (%)**	**N (%)**	**N (%)**	
Presence of inpatient PC consultation services	58 (94)	41 (91)	17 (100)	.57
Presence of dedicated PC outpatient clinic	28 (45)	15 (33)	13 (76)	.**004**
Presence of interdisciplinary SPCT ^b^	38 (61)	31 (69)	7 (41)	.08
Routine identification of PC patients ^c^	36 (58)	22 (51)	13 (76)	.09
Early referral to PC (≥ 3 months)	2 (3)	2 (4)	0 (0)	1.0
Presence of didactic palliative care curriculum^d^	59 (95)	42 (93)	17 (100)	.56
**Integration index**^e^ *(Mean, SD)*	3.6 (.93)	3.4 (.97)	3.9 (.90)	.06

^a^Referral rate: N^o^ of annual inpatient referrals / N^o^ of total annual hospital admissions × 100. Low referral rate < 1%, high referral rate ≥ 1%. ^b^: team of a physician, a nurse and a psychosocial team member (psychologist / counsellor, chaplain, social worker); ^c^ assessment tools for identification of palliative care phase. ^d^ Education provided to nurses, interns, residents and / or fellows hospital-wide. ^e^This represents a composite score of 6 integration indicators; 1 point was given for each affirmative response. Total score ranges from 0 to 6, with a higher index indicating a greater level of integration

## Discussion

This cross-sectional survey shows that the palliative care programmes of almost all hospitals in the Netherlands consist of SPCTs providing inpatient consultation services. Moreover, nearly two third of these SPCTs are interdisciplinary staffed, half of the programmes provide outpatient clinics and a substantial part has dedicated acute care beds and provides community-based palliative care. However, the median referral rate is limited to 0.56% of total annual hospital admissions and referral to these SPCTs occurs late in the disease trajectory.

SPCTs with a high referral rate seem to be more mature than low referral rate SPCTs as the latter frequently have a shorter time of existence and limited staffing with a more basic level of education. In addition, high referral rate SPCTs appear to be better integrated as they are more often related to presence of dedicated outpatient clinics and subsequent earlier timing of referrals, more frequently participate in other departments’ multidisciplinary team meetings and in research, and more often provide education outside their own hospital.

Overall, our three-yearly surveys show that the number of Dutch hospitals providing a SPCT with inpatient consultation services has grown steadily from 39% in 2013, 77% in 2015 to 94% in 2018 [21, 27]. A similar, more gradual pattern exists in the development of dedicated outpatient clinics; 11% of hospitals in 2013, 22% in 2015 and 45% in 2018 [21, 27]. This swift development of palliative care programmes appears a direct result of national professional oncology standards, issued in 2014 and stating that in 2017 a SPCT should be available in every hospital providing cancer care [20].

Despite these developments, clearly there are areas for improvement. A one-day observational study in 14 Belgian hospitals has previously demonstrated that almost 10% of the admitted population were patients in a palliative care trajectory and one third of them had a life expectancy shorter than 3 months [28]. Moreover, in high income countries it has been estimated that 30–45% of palliative care needs may require specialist palliative care [29–31]. These data seem to indicate that hospital referral rates to specialist palliative care could be expected to approximate 3–4%. This indication is supported by results from the National Palliative Care Registry in the United States (US) demonstrating a steadily increasing overall referral rate from 2.5% in 2008 to 5.3% in 2017 [19]. In comparison, although the mean specialist palliative care referral rate in Dutch hospitals increased from 0.6% in 2015 to 0.85% in 2018 [21], service penetration for patients in a palliative care trajectory appears low.

Comparing the integration indicators from our study to a similar survey among 152 ESMO-DCs of integrated Oncology and PC across the world [14], shows that results for presence of inpatient consultation services are alike, respectively 94 and 90%. Results differ for the presence of outpatient PC clinics (45% vs. 89%), interdisciplinary staffing (61% vs 95%), and didactic palliative care curriculum (95% vs 52%). Regarding timing of referral, most referrals in this ESMO survey occurred between 40 and 150 days before death for outpatients and between 14 and 45 days for inpatients, whereas only 3 % of our hospitals refer most patients with specialist palliative care needs earlier than 90 days before death. The authors concluded that ESMO-DCs of Integrated Oncology and PC had high levels of palliative care programme organization, but clinical processes related to timing of referral and education remained areas for further development [14, 32]. From our results inpatient consultation services, interdisciplinary staffing and didactic palliative care curricula seem well embedded in most hospital palliative care programmes. However, provision of outpatient clinics and timing of referral seem to warrant further development.

Considering how to improve referral rate and timing of referral, our study shows that high referral rates were related to staffing of SPCTs and their level of education. This finding is in line with other international studies. A survey focusing on integration of palliative care and oncology among 183 institutions across the world noted that a lack of adequately trained palliative care physicians and nurses was one of the most common barriers to palliative care access and development [33]. The previously mentioned ESMO survey underlined this notion by showing that a higher level of education of SPCTs improved integration between specialist palliative care and oncology [14]. Moreover, the US Palliative Care Registry demonstrated increased referral rates were associated with higher staffing levels, which were subsequently associated with earlier initial palliative care consultation during hospital admission [18].

In addition, our results show that high referral rate SPCTs provided dedicated outpatient clinics significantly more frequently than low referral rate SPCTs and their referrals occurred earlier in the disease trajectory. A recent population-based study in the Netherlands showed that patients provided with palliative care more than 30 days before death were 5 times less likely to experience potentially inappropriate end-of-life care than those with palliative care in the last 30 days or not at all [9]. From our results for timing of referrals it would therefore appear that high referral rate SPCTs may provide better quality of end-of-life care than low referral rate SPCTs.

Multiple randomized controlled trials have demonstrated a positive impact of specialist palliative care on quality of life and quality of end-of-life care when provided in outpatient settings rather than inpatient settings [26, 34] and when provided early and systematically [1, 2, 5, 35]. Moreover, formal screening criteria or palliative care triggers supporting generalist palliative care professionals to select patients for referral, were significantly associated with higher referral rates [19]. Late referrals or a wish to increase referrals were the most commonly cited reasons for implementation [36].

Based on international literature we hypothesized that hospital palliative care programmes with high referral rates would be better staffed and better integrated, with earlier timing of referrals.

Using referral rate and 6 integration indicators our hypothesis was confirmed with regard to staffing and timing of referral and nearly confirmed with regard to level of integration. Moreover, analysing the characteristics related to high referral rate and integration enabled us to identify areas that could improve availability and accessibility to specialist palliative care.

Based on these results we consider referral rate and integration indicators to be useful assets for our next survey.

### Strengths and limitations

This nationwide survey provides a unique insight into the development of SPCTs in Dutch hospitals and characteristics associated with high referral rate. A strength of this study is the high response rate (81%) and the non-responders information indicating that the non-responders had similar characteristics with regard to hospital type, ESMO-DC certification and presence of an operational SPCT. Therefore selection bias is unlikely to have occurred and our findings seem generalizable to all Dutch hospitals. However, some limitations need mentioning.

Although referral rate is an objective measure of accessibility and availability of specialist palliative care and our results are in line with international studies, (trends in) referral rates must be interpreted cautiously. They are likely to be influenced by differences in patient populations (i.e. case-mix) and changes in characteristics of the hospital population over time such as age, disease burden or patterns of diagnoses [37, 38]. Ideally, when comparing referral rates, these factors should be taken into account.

The use of international integration indicators made it possible to compare our results to international research. In addition, it enabled us to compare integration of palliative care programmes with high referral rate SPCTs and low referral rate SPCTs. However, not all major integration indicators were available from our data and some indicators had to be adjusted. For future reference our survey for hospital palliative care programmes may be more specifically tuned to the 13 major integration indicators suggested in the literature to fully assess their usefulness for evaluation of hospital-wide integration of specialist palliative care programmes [13].

A final limitation of this study is the self-reporting nature of the questionnaire. Not all data were necessarily quantified on a patient-level as not all SPCTs register all requested information from their consultations. This may potentially have led to reporting bias. Quantifying data of SPCTs and assessing their impact on quality indicators for end-of-life care in the Netherlands is currently subject of further research [39, 40].

### Conclusion and policy implications

Almost all hospitals in the Netherlands have a palliative care programme with a specialist palliative care team. While they have varying levels of integration and development, and more mature teams show higher referral rates, referral of patients with specialist palliative care needs mostly occurs too little and frequently too late. To improve availability of specialist palliative care for support of patients and generalist palliative care professionals, hospitals may consider appropriately staffing and training their SPCTs and implementing palliative care triggers for referral. Adding dedicated outpatient clinics to inpatient consultation services can contribute to early accessibility of SPCTs. On a smaller scale, SPCTs may consider participation in other departments’ multidisciplinary team meetings, education in the community and participation in research to increase their service penetration. To support this development on a national level, extending professional oncology standards with these recommendations may prove to be a strong incentive.

## Supplementary Information

**Additional file 1.** Specialist palliative care team (SPCT) in hospitals national survey – 2018. Questionnaire.

## Data Availability

The datasets generated and analysed during the current study are held securely by the Netherlands Comprehensive Cancer Organisation and are not publicly available due to confidentiality but are available from the corresponding author on reasonable request.

## Abbreviations

SPCT: specialist palliative care team
ESMO: European Society for Medical Oncology
ESMO - DCs: ESMO**-**Designated Centres
PC: Palliative Care
STROBE: Strengthening the Reporting of Observational Studies in Epidemiology
IQR: Interquartile range
CME: Continuing medical education
CNE: Continuing nursing education
MDTMs: Multidisciplinary team meetings
US: United States
