# Supplementary material for: Specialist palliative care teams and characteristics related to referral rate: a national cross-sectional survey among hospitals in the Netherlands
Source: BMC Palliat Care. 2021 Nov 11;20:175. doi: 10.1186/s12904-021-00875-3 (PMC8582112; doi:10.1186/s12904-021-00875-3)
Supplement: Supplementary file 1 — Additional file 1. Specialist palliative care team (SPCT) in hospitals national survey – 2018. Questionnaire. [file 12904_2021_875_MOESM1_ESM.pdf]

Specialist palliative care teams (SPCT) in hospitals, national survey questionnaire – 2018

Netherlands Comprehensive Cancer Organisation (IKNL), Utrecht, Netherlands & Erasmus MC,

University Medical Center Rotterdam, Department of Public Health, Rotterdam, the Netherlands

<u>I. General questions regarding characteristics and organization of palliative care in your hospital</u>

1. What is the name of the hospital?
2. What is your position within the hospital?
3. What was the total number of hospital admissions in 2017?
   - o Total number of hospital admissions:…
4. Type of hospital
   - o General hospital
   - o Teaching hospital
   - o University hospital
   - o Oncological centre
5. Was a specialist palliative care team operational in the hospital in 2017?
   - o Yes
   - o No
   - o No, specialist palliative care team is still in formation.
     Expected date of start:… *(dd/mo/yy)*
6. Is there an outpatient clinic for palliative care?
   - o Yes
   - o No
7. Is there a daycare unit for palliative care?
   - o Yes
   - o No
8. Are there labeled beds for palliative care?
   - o Yes
   - o No
9. Are the labeled beds concentrated on a designated palliative care unit?
   - o Yes
   - o No
10. Are there wards with nurses that have palliative care as their special field of interest and education?
    - o Yes
    - o No
11. Which wards work with nurses that have palliative care as their special field of interest and education?

o Cardiology	o ICU	o Pediatrics
o Dermatology	o Internal diseases	o Psychiatrics
o Ear, Nose, Throat	o Neonatal care	o Pulmonary diseases
o Emergency Department	o Neurology	o Rehabilitation
o Endocrinology	o Neurosurgery	o Rheumatology
o Gastro-intestinal diseases	o Nephrology	o Surgery
o Geriatrics	o Oncology	o Urology
o Gynecology	o Ophthalmology	
o Hematology	o Orthopedic Surgery	o Other, namely:…

12. What is the hospital’s policy with regard to palliative care? (multiple boxes may be ticked)
- No policy
- Assignment from the board of directors or medical staff to develop palliative care
- Palliative care program / specialist palliative care team with its own multi-year strategy
- Palliative care integrated in the hospital’s multi-year strategy
- A palliative care committee or steering group
- A palliative care faculty
- Other, namely:…

13. Next to the specialist palliative care team, is there a separate pain team operational?
- Yes
- No

14. Are tools / measurement instruments being used within the hospital for identification of palliative care patients?
- Yes
- No

15. What tools / measurement instruments are used to identify palliative care patients?
- SPICT (Supportive and Palliative care IndiCators Tool)
- RADPAC (RADboud indicators for PAlliative Care needs)
- Surprise Question
- Other, namely:…

II. General questions regarding characteristics and organization of the specialist palliative care team (SPCT)

16. What is your position within the SPCT?
- Head of SPCT / Palliative care program leader
- Team member
- Other, namely:…

17. When did the team start? (dd/mo/yy)

18. What was the number of inpatient consultations for the SPCT in 2017?
- N.a.; SPCT does not provide inpatient consultation services
- Number of inpatient consultations: …

19. What was the number of outpatient consultations for the SPCT in 2017?
- N.a.; SPCT does not provide outpatient consultation services
- Number of outpatient consultations: …

20. What was the number of home visits of the SPCT in 2017?
- N.a.; SPCT does not provide home visits
- Number of home visits: …

21. Which disciplines are represented in the SPCT ? (multiple boxes may be ticked)

o General practitioner	o Radiotherapy	o Pharmacy
o Nursing home physician	o Rehabilitation	o Psychiatry
o Anesthesiology	o Nurse practitioner	o Psychology
o Internal diseases (oncology)	o Oncology nurse	o Social worker
o Gastro-enterology	o Pain nurse	o Occupational therapy
o Geriatrics	o Community nurse	o Physical therapy
o Pediatrics	o Hospital nurse	o Chaplains
o Pulmonary diseases	o Transfer nurse	o Dietician
o Neurology	o Other nurse	o Secretary
o Other, namely:…		

22. Which discipline / specialty is head / coordinator of the SPCT? (multiple boxes may be ticked)

o General practitioner	o Radiotherapy	o Pharmacy
o Nursing home physician	o Rehabilitation	o Psychiatry
o Anesthesiology	o Nurse practitioner	o Psychology
o Internal diseases (oncology)	o Oncology nurse	o Social worker
o Gastro-enterology	o Pain nurse	o Occupational therapy
o Geriatrics	o Community nurse	o Physical therapy
o Pediatrics	o Hospital nurse	o Chaplains
o Pulmonary diseases	o Transfer nurse	o Dietician
o Neurology	o Other nurse	o Secretary
o Other, namely:…		

23. Which disciplines are not represented on the SPCT but are closely affiliated for consultation? (multiple boxes may be ticked)

o Internal diseases (oncology)	o Radiotherapy	o Psychiatry
o Anesthesiology	o Gastro-enterology	o Chaplains
o Neurology	o Pharmacy	o Social worker
o Pulmonary diseases	o Psychology	o Other, namely:…

24. For each SPCT member please indicate discipline / medical specialty and the number of labeled hours per week. In addition, specify the extra efforts (in hours) for the SPCT, if applicable.
(The online questionnaire allowed multiple entries per discipline)

	Labeled hours / week	Average extra effort (hrs /w)
○ General practitioner	○	○
○ Nursing home physician	○	○
○ Anesthesiology	○	○
○ Internal diseases (oncology)	○	○
○ Gastro-enterology	○	○
○ Geriatrics	○	○
○ Pediatrics	○	○
○ Pulmonary diseases	○	○
○ Neurology	○	○
○ Radiotherapy	○	○
○ Rehabilitation	○	○
○ Nurse practitioner	○	○
○ Oncology nurse	○	○
○ Pain nurse	○	○
○ Community nurse	○	○
○ Hospital nurse	○	○
○ Transfer nurse	○	○
○ Other nurse	○	○
○ Pharmacy	○	○
○ Psychiatry	○	○
○ Psychology	○	○
○ Social worker	○	○
○ Occupational therapy	○	○
○ Physical therapy	○	○
○ Chaplains / spiritual caregivers	○	○
○ Dietician	○	○
○ Secretary	○	○
○ Other, namely…	○	○

<u>III. Questions regarding financing of the SPCT</u>

25. How is the SPCT financed? (multiple boxes may be ticked)
- ○ From patients' DBC (Diagnosis-Treatment Combination) palliative care (healthcare insurance)
- ○ Own financial means from the hospital
- ○ Otherwise, namely:

26. Which medical specialty can initiate a DBC palliative care? (multiple boxes may be ticked)
- ○ Anesthesiology
- ○ Internal diseases (oncology)
- ○ Geriatrics
- ○ Pulmonary diseases
- ○ Neurology
- ○ Pediatrics

27. Are SPCT consultations registered?
- ○ Yes
- ○ No

28. Are registered SPCT consultations billed to patients' health insurances?
   - <20% of registered consultations is billed
   - 20-40% of registered consultations is billed
   - 40-60% of registered consultations is billed
   - 60-80% of registered consultations is billed
   - > 80% of registered consultations is billed
   - No, registered consultations are not billed, because…

29. Are there any contracts with health insurance companies about reimbursement?
   - Yes
   - No

30. Are there internal agreements about reimbursement of the SPCT with the board / medical staff?
   - Yes
   - No

31. Was the number of consultations in 2017 in accordance with the number of labeled hours for the members of the SPCT?
   - No, our SPCT actually had too many consultations
   - No, our SPCT actually had too few consultations
   - Yes, the number of consultations was in line with the number of labeled hours

<u>IV. Questions regarding procedures followed by the SPCT</u>

32. Who can request consultation from the SPCT?

○ Medical specialist	○ General practitioner	○ Paramedics
○ Resident	○ Pharmacist	○ Patient / family
○ Intern	○ Nurse	○ Primary healthcare professionals
○ Other, namely…	○	○

33. Are there triggered referrals to the SPCT for patients with specific diagnoses?
   - No
   - Yes, namely…

34. What is the average life expectancy for patients referred to the SPCT?
   - 3 days or less
   - 4 days – 2 weeks
   - 2 – 4 weeks
   - 4 weeks – 3 months
   - 3 months or more
   - Unknown

35. For which type of patients can the SPCT be consulted? (multiple boxes may be ticked)
   - Inpatients
   - Outpatients
   - Patients who live at home (or elsewhere) and are known to the SPCT
   - Patients who live at home (or elsewhere) and are not necessarily known to the SPCT
   - Others, namely:

36. How does the request for consultation reach the SPCT? (multiple boxes may be ticked)
   - By phone
   - Via the electronic patient record
   - Via a paper referral form
   - Via the multidisciplinary team meeting
   - Otherwise, namely:

37. Which types of consultation are provided **to patients**? (multiple boxes may be ticked)
    - By telephone
    - Bedside (inpatient)
    - Face to face (outpatient)
    - Home visit
    - Screen tot screen
    - By e-mail
    - Otherwise, namely:…
38. Which types of consultation are provided **to professionals**? (multiple boxes may be ticked)
    - By telephone
    - Face to face
    - Screen tot screen
    - By e-mail
    - Otherwise, namely:…
39. How is the referring professional provided with the SPCT's advice? (multiple boxes may be ticked)
    - By telephone
    - Face to face with referring professional
    - Screen tot screen with referring professional
    - In the electronic patient record
    - In the paper patient file
    - On the paper referral form
    - Otherwise, namely:…
40. Where is the consultation documented? (multiple boxes may be ticked)
    - In the electronic patient record
    - In the paper patient file
    - In the SPCT's own patient database
    - In the patient's individual care plan
    - Otherwise, namely:…
41. At what times can the SPCT be consulted?
    - 24 / 7
    - Within office hours
    - Otherwise, namely:
42. Is consultation available outside office hours?
    - Yes, through SPCT medical specialist on call
    - Yes, via answering machine / e-mail
    - Yes, through a regional SPCT helpdesk (by phone)
    - Yes, through supraregional SPCT cooperation
    - No, consultation outside office hours is not availble
    - Otherwise, namely:…
43. How much time is available for the initial (first) consultation (in minutes)?

    ……………………….
44. How much time is available for follow-up consultation (in minutes)?

    ……………………….

45. **As a rule**, is the patient seen by one or more members of the SPCT?
   - No, usually the consultation is done by telephone with the referring professional
   - As a rule, the patient is seen by a nurse practitioner
   - As a rule, the patient is seen by a nurse
   - As a rule, the patient is seen by a physician
   - As a rule, the patient is seen by a nurse practitioner **and** a physician
   - As a rule, the patient is seen by a nurse **and** a physician
   - As a rule, the patient is seen by a nurse practitioner **or** a physician
   - As a rule, the patient is seen by a nurse **or** a physician
   - As a rule, the patient is seen by a nurse **or** nurse practitioner
   - Otherwise, namely:…
46. What is the average number of contacts of the SPCT with the patient?
   - Only once (initial visit)
   - Daily SPCT visits during hospital admission
   - Otherwise, namely:…
47. What are reasons for referral to the SPCT? (Rank the list from highest to lowest frequency)
   - Explaining illness and prognosis / prognostic awareness
   - Problems / symptoms within the physical domain
   - Problems / symptoms within the psychological domain
   - Problems / symptoms within the social domain
   - Problems / symptoms within the spiritual / existential domain
   - Weighing treatment options
   - Coping of patient and / or family
   - Advance care planning
   - Referral
   - Palliative sedation
   - Euthanasia
48. Are there other reasons for referral to the SPCT?
   - Yes, namely:…

49. Which of the following measurement instruments / tools are used in daily practice?
(multiple boxes may be ticked)

- o Karnofsky Performance Status (KPS)
- o Palliative Performance Status (PPS)
- o Eastern Cooperative Oncology Group Performance Status (ECOG)
- o Medical Research Council (MRC) Dyspnea
- o Clinical COPD Questionnaire (CCQ)
- o Mouth status screening list
- o Oral Mucositis Assessment Scale (OMAS)
- o Mini-Nutritional Assessment-Short Form (MNA SF)
- o Short Nutritional Assessment Questionnaire (SNAQ)
- o Malnutrition Universal Screening Tool (MUST)
- o Pain Inventory
- o Breakthrough Pain Assessment Tool (BAT)
- o Rotterdam Elderly Pain Observation Scale (EPOS)
- o Pain Assessment Scale for Seniors with severe dementia (PACSLAC-D)
- o Brief Fatigue Inventory (BFI)
- o Other, namely:…
- o Multidimensional Fatigue Index (MFI)
- o Distress Thermometer
- o Edmonton Symptom Assessment Scale (ESAS)
- o Chronic Respiratory Questionnaire (CRQ)
- o Care related Quality of Life for Chronic Heart Failure (CareQoL CHF)
- o Groningen Frailty Indicator (GFI)
- o Geriatric 8 (G8)
- o Hospital Anxiety and Depression Scale (HADS)
- o Cornell Scale for Depression in Dementia (CSDD)
- o Geriatric Depression Scale 15 (GDS15)
- o Delirium Observation Scale (DOS)
- o Delirium-O-Measure (DOM)
- o Pain Assessment in Advanced Dementia (PAINAD)
- o Experienced Burden by Informal Caregiver (EDIZ)
- o Self-rated Burden Scale (SRB)
- o We do not use measurement tools

50. What is the SPCT physician's position with regard to referred patients?

- o Managing / Treating Physician
- o Co-managing Physician
- o Consulting Physician

51. What proportion of referrals concerned non-oncology patients?

- o < 20%
- o 20-40%
- o 40-60%
- o 60-80%
- o > 80%

V. Coordination & continuity of care

52. Prior to discharge, is the SPCT in touch with the nursing home physician or general practitioner, regarding palliative care at home?

- o Yes, always
- o Only on indication
- o No, that is the responsibility of the managing / treating physician
- o No, because…

53. The following questions concern SPCT continuing advice / counseling after the patient has been discharged. (multiple boxes may be ticked)

	Always	Often	Sometimes	Never
Is there follow-up by phone?	o	o	o	o
Is there follow-up in the outpatient clinic?	o	o	o	o
Is there follow-up through community visits, if needed?	o	o	o	o
Follow-up is provided in other ways, namely:…				

54. Our SPCT's community-based way of working is apparent through: (multiple boxes may be ticked)
- We do not work community-based
- Composition of the SPCT with professionals from both hospital and community setting
- Consultation by phone for professionals caring for patients outside the hospital
- Bedside consultation by SPCT members for patients outside the hospital
- Community based healthcare professionals perform bedside consultation in hospital
- Community based healthcare professionals participate in SPCT's multidisciplinary team meeting
- Otherwise, namely:

55. What activities does the SPCT perform for the purpose of publicity and profiling? (multiple boxes may be ticked)
- Research
- Education / training (within the hospital)
- Education / training (outside the hospital)
- Development of protocols
- PR / marketing
- Initiating presentations, conferences, congresses
- Otherwise, namely:…

<u>VI. SPCT Multidisciplinary team meeting</u>

56. Is there a weekly multidisciplinary team meeting (MTM) of the SPCT?
- Yes
- No, our MTM frequency is:…. times per …

57. What is the average duration of the MTMs? (in minutes)…

58. Which referring disciplines attend the MTMs? (multiple boxes may be ticked)

	Standing invitation	When indicated
○ General practitioner	○	○
○ Nursing home physician	○	○
○ Anesthesiology	○	○
○ Internal diseases (oncology)	○	○
○ Gastro-enterology	○	○
○ Geriatrics	○	○
○ Pediatrics	○	○
○ Pulmonary diseases	○	○
○ Neurology	○	○
○ Radiotherapy	○	○
○ Rehabilitation	○	○
○ Nurse practitioner	○	○
○ Oncology nurse	○	○
○ Pain nurse	○	○
○ Community nurse	○	○
○ Hospital nurse	○	○
○ Transfer nurse	○	○
○ Other nurse	○	○
○ Pharmacy	○	○
○ Psychiatry	○	○
○ Psychology	○	○
○ Social worker	○	○
○ Occupational therapy	○	○
○ Physical therapy	○	○
○ Chaplains / spiritual caregiver	○	○
○ Dietician	○	○

- Secretary ○ ○
- Other discipline, namely…

59. Does the referring professional attend the MTM?
    - Yes, in principal the referring professional always attends the MTM
    - Only when indicated
    - No
60. Which patients are discussed in the MTMs?
    - All patients
    - Only new patients
    - Only complex patients
    - Only new and complex patients
    - Others, namely…
61. How many patients are on average discussed in the MTMs?...
62. Does the referring professional always receive a report of the MTM?
    - Yes
    - No
63. Does the general practitioner or nursing home physician of the referred patient always receive a report of the MTM?
    - Yes
    - No
64. Does a member of the SPCT attend MTMs in other departments?
    - Yes
    - No
65. The SPCT has a standing invitation to attend the MTMs of the following departments: (multiple boxes may be ticked)

○ Anesthesiology	○ Hematology	○ Orthopedic Surgery
○ Cardiology	○ ICU	○ Pediatrics
○ Dermatology	○ Internal diseases	○ Psychiatrics
○ Ear, Nose, Throat	○ Neonatal care	○ Pulmonary diseases
○ Emergency Department	○ Neurology	○ Rehabilitation
○ Endocrinology	○ Neurosurgery	○ Rheumatology
○ Gastro-intestinal diseases	○ Nephrology	○ Surgery
○ Geriatrics	○ Oncology	○ Urology
○ Gynecology	○ Ophthalmology	○ Other, namely:…

<u>VII. Quality of care and expertise of the SPCT</u>

66. Are there agreed quality criteria? (multiple boxes may be ticked))
    - No
    - Yes, regarding timing / response to referral
    - Yes, regarding level of education of SPCT members
    - Yes, regarding method of consultation
    - Yes, regarding advice based on palliative care guidelines
    - Yes, regarding use of measurement instruments / tools
    - Yes, regarding presence at SPCT MTMs
    - Yes, regarding follow-up of given advice
    - Yes, regarding informing patients' general practitioner
    - Yes, regarding collective continuing medical / nursing education
    - Yes, regarding 'care for healthcare professionals'
    - Other criteria, namely:

67. What is the level of education and / or training of the physicians of the SPCT?

	Number of physicians
○ 2- year continuing medical education (CME)	…
○ Cardiff Palliative Medicine Course (postgraduate)	…
○ 8-day medical course	…
○ No additional education and / or training	…
○ Other, namely:	…

68. What is the level of education and / or training of the nurses of the SPCT?

	Number of nurses
○ 1-year continuing nursing education (CNE) Nijmegen	…
○ 1-year continuing nursing education (CNE) Rotterdam	…
○ 1-year continuing nursing education (CNE) Utrecht	…
○ Basic palliative care training	…
○ No additional education and / or training	…
○ Other, namely:	…

69. What is the level of education and / or training of the nurse practitioners of the SPCT?

	Number of nurses
○ Differentiation Palliative Care	…
○ Differentiation Oncology	…
○ Differentiation Pain	…
○ No additional education and / or training	…
○ Other, namely:	…

70. Which members of the SPCT are reimbursed for attending conferences and / or continuing medical / nursing education? (multiple boxes may be ticked)

○ General practitioner	○ Radiotherapy	○ Pharmacy
○ Nursing home physician	○ Rehabilitation	○ Psychiatry
○ Anesthesiology	○ Nurse practitioner	○ Psychology
○ Internal diseases (oncology)	○ Oncology nurse	○ Social worker
○ Gastro-enterology	○ Pain nurse	○ Occupational therapy
○ Geriatrics	○ Community nurse	○ Physical therapy
○ Pediatrics	○ Hospital nurse	○ Chaplains
○ Pulmonary diseases	○ Transfer nurse	○ Dietician
○ Neurology	○ Other nurse	○ Secretary
○ Other, namely:…		○ No budget

71. Is there structural attention for selfcare and care for each other in SPCT meetings?
- ○ Yes
- ○ No

72. Is there a burn-out (prevention) programme within the hospital and does the SPCT participate?
- ○ Yes
- ○ Yes, but the SPCT does not participate
- ○ No

73. How would you evaluate the over-all functioning of the SPCT within your hospital?

0 50 100
|____|

74. How would you evaluate the quality of consultation provided by the SPCT?

0 50 100
|____|

75. What impeding factors are currently influencing the over-all functioning of the SPCT?...

76. What tips and tricks would you suggest to improve over-all functioning of a SPCT?...

77. May the researchers contact you, should results of the survey so require?
- ○ Yes
- ○ No

Thank you for your cooperation!
